# Surgical Anatomy for Sterilization Procedures in Female Capybaras

**DOI:** 10.3390/ani13030438

**Published:** 2023-01-27

**Authors:** Fabiana M. G. Jorge, Flavia Maria Pia Montenegro Donoso, Mayla Magalhães de Oliveira Alcobaça, Marilu Cristofoli, Fernanda B. Passos Nunes, Cristiane S. Pizzutto, Antonio Chaves de Assis Neto

**Affiliations:** 1 Department of Surgery, School of Veterinary Medicine and Animal Science of São Paulo University, São Paulo 05508-270, Brazil; 2 A Z Nunes & Cia Ltda ME, Itu 13301-521, Brazil; 3 Department of Reproduction, School of Veterinary Medicine and Animal Science of São Paulo University, São Paulo 05508-270, Brazil; 4 UniEduk—Unifaj and Unimax, Jaguariúna 13820-000, Brazil; 5 Reprocon, Reproduction for Conservation Research Group, Campo Grande 79052-280, Brazil

**Keywords:** abdominal anatomy, minilaparotomy, population control, reproductive anatomy, rodents

## Abstract

**Simple Summary:**

Capybaras are the largest rodents cohabiting with humans within urban and peri-urban green areas. Surgical contraception has been recommended as a method of population control in cases of overpopulation and/or zoonotic disease transmission; however, scant data are available on sterilization procedures and relevant surgical anatomy. For surgical anatomic description, eight female capybara cadavers were dissected; the layers of the abdominal wall are described as well as the access to the uterine tubes and uterine horns by a lateral or ventral midline approach. For better description of the abdominal wall, ultrasonography was performed in one (1) living female. The uterine tubes were easily accessed by the bilateral flank approach, whereas the uterine horns were more easily exposed by the ventral midline approach. Given the lack of data regarding surgical anatomy for contraceptive surgeries in female capybaras, this study helps achieve more efficient contraceptive surgeries, both reducing the total surgical time and enhancing animal welfare.

**Abstract:**

Capybaras are the largest rodents cohabiting with humans within urban and peri-urban green areas and are known by their prolificity. Surgical contraception has been recommended by official organizations as a way to control capybara populations in areas of zoonotic disease transmission, but little data are available concerning surgical anatomy. To obtain objective anatomical descriptions related to reproductive organs, eight female capybaras cadavers were dissected. The stratigraphy of the lateral (flank) and ventral, post-umbilical (on the linea alba) abdominal wall is described as well as the vascular anatomy of reproductive organs and their syntopy with the abdominal viscera. We commented on the access to the uterine tubes and uterine horns for each approach, and for better description of abdominal wall stratigraphy, abdominal ultrasonography was performed in one live female. All of the animals were provenient from “in situ” population management projects that were properly authorized. Similar abdominal wall stratigraphy was found in comparison to domestic mammals, with emphasis on a thick cutaneous muscle, a thin linea alba, and a large, loose cecum. The uterine tubes were easily accessed by bilateral laparotomy, allowing tubal removal/ligation procedures, while uterine horn exposure was more readily reached by a midline post umbilical celiotomy, favoring horn ligature and hysterotomy techniques. This study can help achieve more efficient contraceptive surgeries in capybaras, reducing the total surgical time and enhancing animal welfare.

## 1. Introduction

Capybaras (*Hydrochoerus hydrochaeris*, Linnaeus 1766) are the largest living rodents found only in the Neotropics [1,2,3]. Their high adaptability to modified environments, with high phenotypic plasticity and genetic variability, may result in the remarkable success of the species in anthropogenic areas [4]. This plasticity allied to their high prolificity, with litter size ranging from 1–7 [2], has turned their coexistence with man into a problem in areas where capybaras are overpopulated and/or involved in zoonotic disease transmission, such as Brazilian spotted fever [5,6,7,8]. In this context, surgical sterilization has been recommended by public environmental organizations with the aim of population control [5].

Contraceptive methods that preserve the gonads are used in free-ranging wildlife population control [6,9,10]. Tube sterilization procedures in capybaras have been reported by Passos Nunes, Yanay, Ferraz, Rodrigues, Passos Nunes, and Yanai, [8,10,11,12], using a lateral (flank) or ventral midline approach (LA). More effective access to uterine tubes (*salpinx*) via a bilateral flank approach was described by Yanai, whereas Passos Nunes [6,8] reported a uterine horn ligature technique using a ventral midline approach (on the linea alba—LA), obtaining good uterine horn exposure and hysterotomy performance in pregnant females. Tubal ligation, tubal transection (flank approach), and ovariohysterectomy (ventral midline approach) were also described by Boulanger [13] for surgical control of white-tailed deer (*Odocoileus virginianus*) populations in the United States, reinforcing both lateral and ventral approaches for contraceptive surgery in wild, free-ranging mammals. The awareness about abdominal anatomy, including muscular stratigraphy and the relation between abdominal viscera is essential for proper surgical planning. Little data are available concerning this relation in capybaras, with the data being limited to digestive anatomy and physiopathology [14,15,16]. Imaging techniques have been useful for non-invasive description of the anatomy. Ultrasonography stands out as an accurate and accessible tool to analyze muscle and tendon tissue [17].

With the purpose of describing surgical abdominal anatomy and the enhancement of surgical efficiency in female capybaras, the lateral (flank) and midline, post umbilical (LA) regions of female capybara cadavers were dissected. Furthermore, ultrasonography was performed on one living female to confirm the presence of a complete linea alba. The abdominal wall stratigraphy was described as well as the vascular anatomy of reproductive organs and the syntopy with the abdominal viscera. We sustained the hypothesis that a bilateral laparotomy provides better access to the uterine tubes and ovaries, while a midline post umbilical celiotomy (LA) is preferred for the uterine body and horns. Our conclusion was that the stratigraphy of the abdominal wall follows the general anatomy described for mammals, with variations in muscular thickness and ease of identification of the linea alba. The loose and large cecum occupies the greatest part of the abdomen and is in syntopy with the ovaries (*ovarium*), uterine tubes (*salpinx*), and uterine horns (*cornu uteri, dextrum et sinistrum*). A bilateral laparotomy or a midline post-umbilical celiotomy provided access to the reproductive organs of capybaras. The dorsal location of the ovary and uterine tubes requires a bilateral minilaparotomy for tubal ligation/removal performance and the easy identification of the uterine horns through a median post-umbilical celiotomy makes this approach better for uterine ligature or hysterotomy performance, with it being an efficient alternative to mass sterilization surgeries when the maintenance of the ovary is recommended.

## 2. Materials and Methods

Dissection of the abdominal region was performed in eight female capybara cadavers weighing between 25–80 kg, provenient from population control projects of free-ranging capybaras, previously authorized by corresponding government agencies (Departamento de Fauna da Secretaria de Meio Ambiente de São Paulo—SMA/SP: 0000032821/2022; 00000069455/2021; SISGEN number: A4C6059), and by the ethical committee of the School of Veterinary Medicine and Animal Science of São Paulo University (CEUA—FMVZ/USP: 3990010822). The animals died from different causes not related to management involving trauma and conspecific fighting. All of the cadavers were frozen and thawed in a tank with tepid water prior to dissection. One cadaver was fixed following the Tamayo–Arango technique [18]; for an estimated 65 kg capybara, 15 L of solution was perfused and the fluid overflowed the abdominal tissues. Another cadaver was injected with latex (formula: (C5H8)n, Duo latex brand)) dyed with blue ink for venous highlight (Xadrez line, Sherwin Williams), injected in the caudal direction of the caudal *vena cava*, and red ink for the arteries (Xadrez line, Sherwin Williams), injected in the caudal direction of abdominal aorta. With the objective of enhancing the color and assisting the identification of the cutaneous muscle, *musculus panniculus carnosus,* the third cadaver was inked with a homemade alcoholic reduction of *Hibiscus* sp. (50 g of dry *Hibiscus* sp. and 150 mL of ethanol 70%—formula: C2H5OH, Prolink brand). The other five cadavers were dissected after thawing was complete.

In one adult living female (65 kg) provenient from a research project authorized by government authorities and by the ethical committee of the same institution (CEUA—FMVZ/USP: 1575300621; SISBIO: 81707-1), a ventral abdominal ultrasound was performed objectivating a non-invasive anatomic description of the ventral abdomen (ultrasound device LOGIC E VET, General Electric, USA, micro-convex probe, 7.5 megahertz). The images were made at a transverse plane covering the pre-umbilical (4 cm cranial to the umbilical scar), umbilical, and post-umbilical regions (from the umbilical scar to the pubis).

For a lateral abdominal dissection, the cadaver was set up on a lateral recumbency. The place of dissection was defined utilizing the ovary localization described by Pradere [19] as a base, with a minilaparotomy incision (2.5–3.5 cm) over the cranio–caudal (longitudinal) axis made at the sublumbar region, caudal to the last rib and ventral to the lumbar musculature, near the third intertransverse space. For the ventral abdominal approach, with the cadaver on a dorsal recumbency, a midline minilaparotomy incision (2.5–3.5 cm on LA) was performed, 2 cm cranial to the pubis until the umbilical scar, over the cranio–caudal axis (post-umbilical region). After minilaparotomy incisions, the abdomen was open from the xiphoid to the pubis to allow better description of the visceral syntopy. The stratigraphy of the abdominal wall and ease of access to the uterine tubes and uterine horns was described for each approach as well as the vascular anatomy of reproductive organs and their syntopy with the other organs of the abdominal cavity. The anatomical terms follow the Nomina Anatomica Veterinária [20].

## 3. Results

### 3.1. Lateral Abdominal (Flank) Dissection

**Figure 1:**
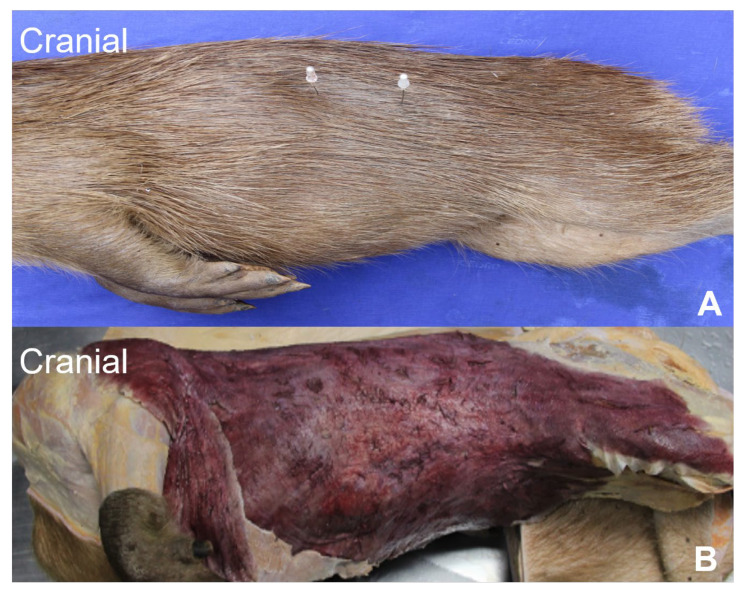
(**A**). Region of dissection of the lateral abdomen (between needles). (**B**). Cutaneous muscle highlighted with hibiscus ink.

The stratigraphy of the lateral abdominal wall at the flank region encompassed the skin, with a thick *dermis* and dermal white adipose tissue (WAT) (Figure 1A), followed by the cutaneous muscle, a thick dermal striated muscle with longitudinal fibers that range from the thorax to the pelvic members (Figure 1B).

**Figure 2:**
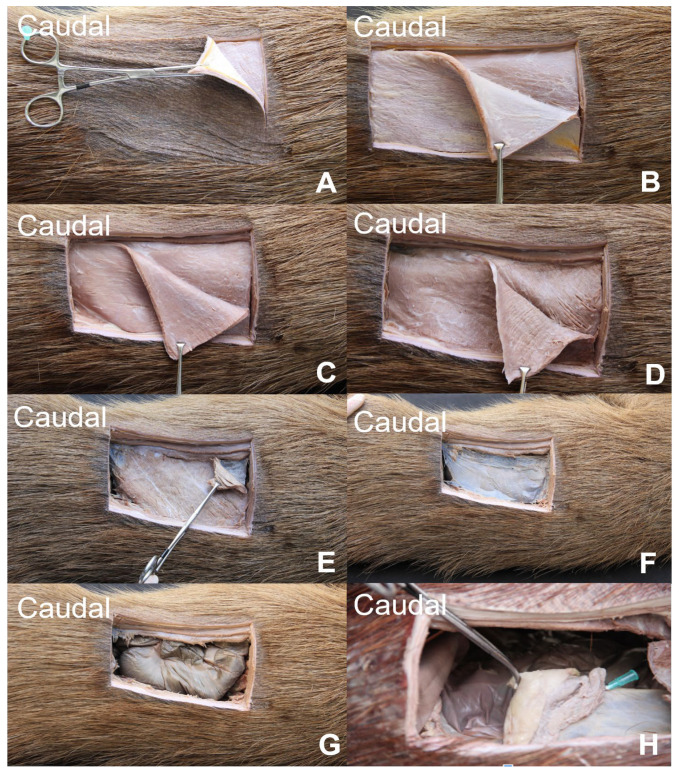
Dissection of left flank region between the last rib and iliac crest; caudal to the left. (**A**) Skin; (**B**) cutaneous muscle being deflected; (**C**) external abdominal oblique muscle being deflected, exposing the internal abdominal oblique muscle; (**D**) internal abdominal oblique muscle being deflected exposing the transversus abdominis muscle; (**E**) transversus abdominis muscle being deflected exposing the parietal peritoneum (**F**); (**G**) cecum covering the ovary and uterine tube; and (**H**) uterine tube and mesosalpinx (green needle).

Subsequently the following structures were dissected: the transverse fascia, external abdominal oblique muscle, internal abdominal oblique muscle (narrow), transversus abdominis muscle (showing slightly oblique fibers), and parietal peritoneum (Figure 2).

The ipsilateral ovary and correspondent uterine tube, located caudally to the caudal pole of the kidney, could be accessed through a minilaparotomy approach with an incision over the cranio–caudal axis (longitudinal) at a lateral recumbency. This approach granted easy exposure of the ipsilateral ovary and uterine tube for tubal ligation/removal, but it did not provide enough exposure to the ipsilateral uterine horns for horn ligature or a hysterotomy. The uterine tube is a tubular structure near the mesosalpinx, and its irrigation comes from the tubarian branch of the ovarian artery, itself a direct branch of the abdominal aorta, emerging cranially to the caudal mesenteric artery. Considering the dorsal position of the ovary, the contralateral ovary could not be pulled through the same incision, demanding two lateral abdominal incisions for the bilateral tubal ligation/removal.

The uterine tubes are in syntopy with the ipsilateral ovary and with the cranial end of the ipsilateral uterine horn. On the right antimer, the uterine tube can be in syntopy with the small intestine (jejunum) on a medial surface depending on the cecum and uterus distention and it is in syntopy with the spiral (distal) ansa of the ascending colon on a ventral surface, with it sometimes being able to make contact with the cecum body (*corpus ceci*) when the cecum is tympanic. On the left antimer, it is in syntopy with the descendent colon in a medial surface and with the body of the cecum on a ventral surface.

### 3.2. Ventral Abdominal Post-Umbilical Dissection (LA)

**Figure 3:**
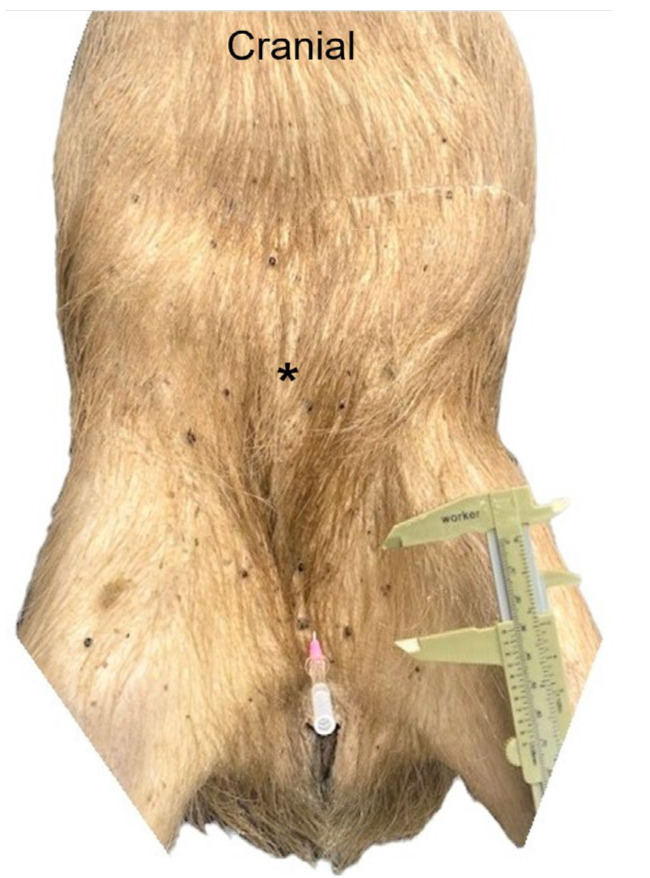
Capybara in dorsal recumbency for ventral abdominal dissection. The dissection started 1 cm cranial to the pubis (pink catheter) until the umbilical scar (asterisk). The incision was extended cranially for description of the abdominal viscera anatomy and syntopy.

**Figure 4:**
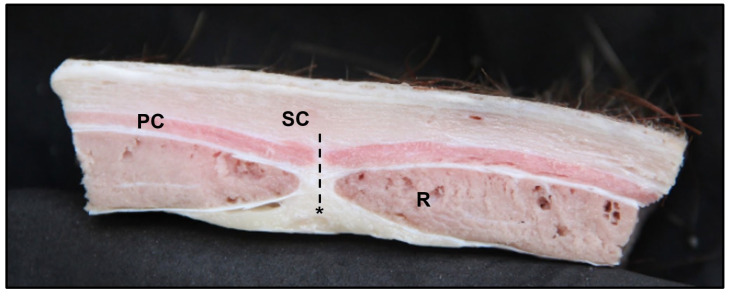
Transverse section of the post-umbilical region. SC: subcutaneous tissue; PC: cutaneous muscle; R: rectus abdominis muscle; dashed line: linea alba; asterisk: adipose tissue.

**Figure 5:**
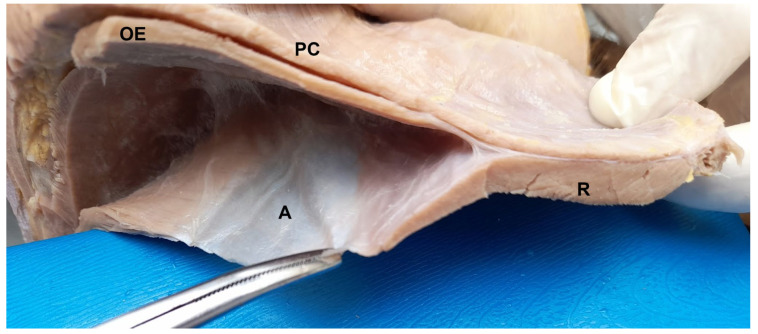
PC: cutaneous muscle; OE: external abdominal oblique muscle; OI: internal abdominal oblique muscle; A: aponeurosis of OI recovering R: rectus abdominis muscle.

The stratigraphy of the abdominal wall at the post-umbilical region encompassed the skin, with the evident cutaneous muscle, the transverse fascia, the aponeurosis, and the flashy part of external abdominal oblique muscle covering the rectus abdominis muscle (*m. rectus abdominis*) in a superficial plane, the aponeurosis of internal abdominal oblique muscle covering the rectus abdominis muscle on a superficial and deep plane, and the aponeurosis of transversus abdominis muscle covering the rectus abdominis muscle in a deep plane, forming together the rectus sheet (*vagina m. recti abdominis*) with its internal and external lamina (*lamina interna* and *lamina externa)*, as well as the *linea alba*, in a median plane, dividing the two muscular bands of the rectus abdominis muscle (Figure 3, Figure 4 and Figure 5).

**Figure 6:**
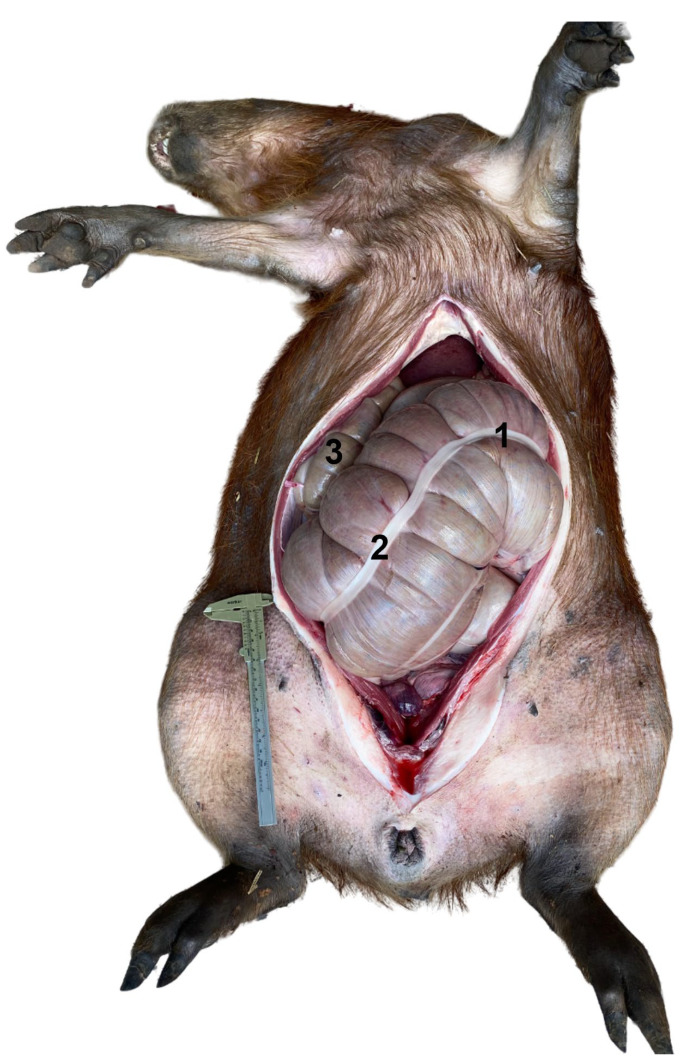
Capybara in dorsal recumbency. Ventral midline incision showing the extension of the cecum when tympanic. (1) Cecum left flexure, in syntopy with the left ovary; (2) cecum body; (3) cecum apex.

Identification of the linea alba is not always easy and the surgeon might have some difficulty in properly locating it. In some dissections, the presence of the *linea alba* could not be confirmed in the post-umbilical region because of its slenderness. The parietal peritoneum is the deepest layer to be incised so the abdominal cavity can be accessed. The first organ to be seen in a ventral view is the urinary bladder (easily pulled out of the cavity when full of urine), followed by the uterine body and uterine horns, both in syntopy with the descending colon (*colon descendens*). There is no evident great omentum supporting the abdominal organs at the most ventral view and the body of the cecum is found cranially to the urinary bladder. The cecum can occupy the pre-umbilical, umbilical, and post umbilical regions depending on its distention by ingesta and gas (Figure 6). The basis of the cecum (*basis ceci*) is at the median plane of the abdominal cavity and is attached to the mesentery root (*radix mesenterii*) by a peritoneum fold, allowing the body of the cecum to be displaced to the left or to the right depending on the uterine distention in pregnant females. The cecum base gives rise to a sacculation where the cecum body begins. From the cranial part of this sacculation, the proximal ansa of the ascending colon begins and follows alongside the cecum body attached to it by the cecocolic fold (*plica cecocolica*). The cecum body goes on a superficial plane to the left side of the abdomen, making a flexure on the left flank. It returns to the middle abdomen and continues to the right, taking a dorsal direction to finish again in the middle plane with its apex (*apex ceci*), attached by the pyloric region through a peritoneal fold. The colic duodenal fold is present as well (*plica duodenocolica*).

**Figure 7:**
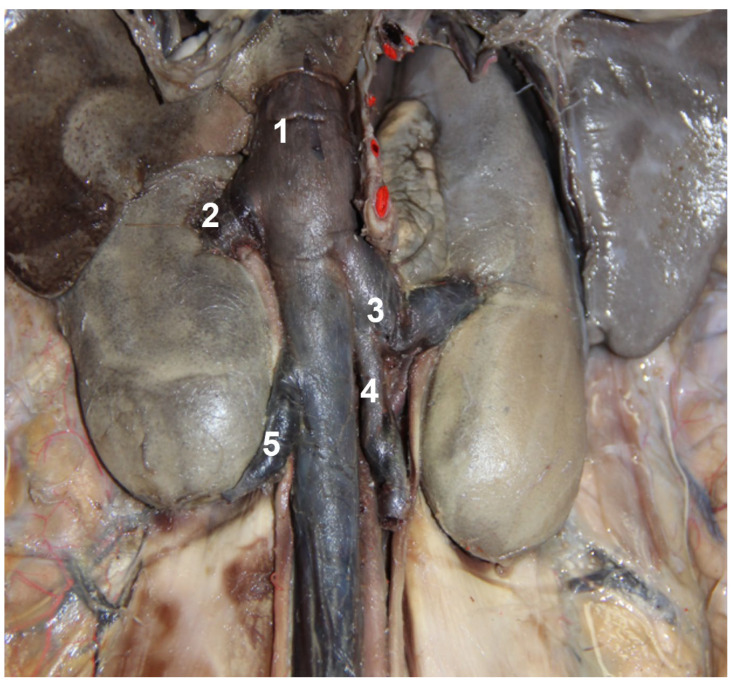
Topographic region of the kidneys. Viscera removed to show vascular distribution: 1. caudal vena cava receiving right ovarian vein (5); 2. right renal vein; 3. left renal vein receiving left ovarian vein (4).

**Figure 8:**
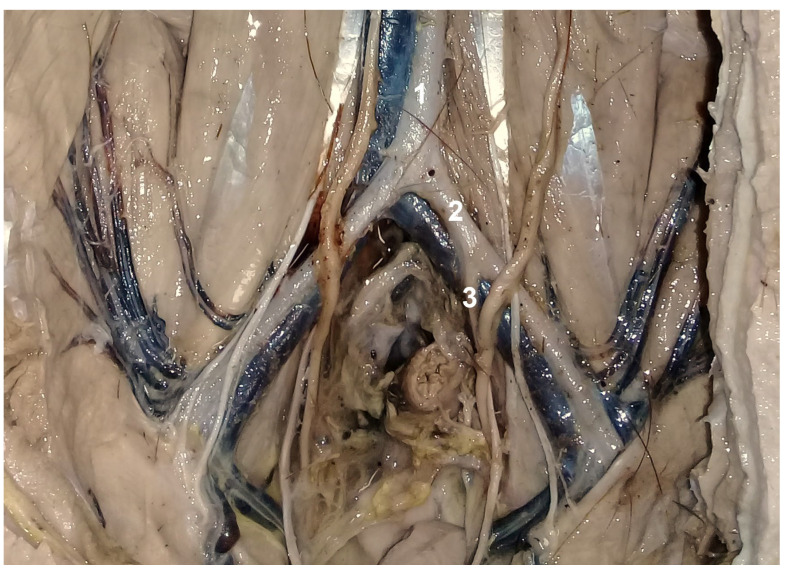
Topographic region of pelvis. Viscera removed to show vascular distribution: 1. Aorta; 2. common iliac; 3. internal iliac, that will branch to become the uterine artery.

**Figure 9:**
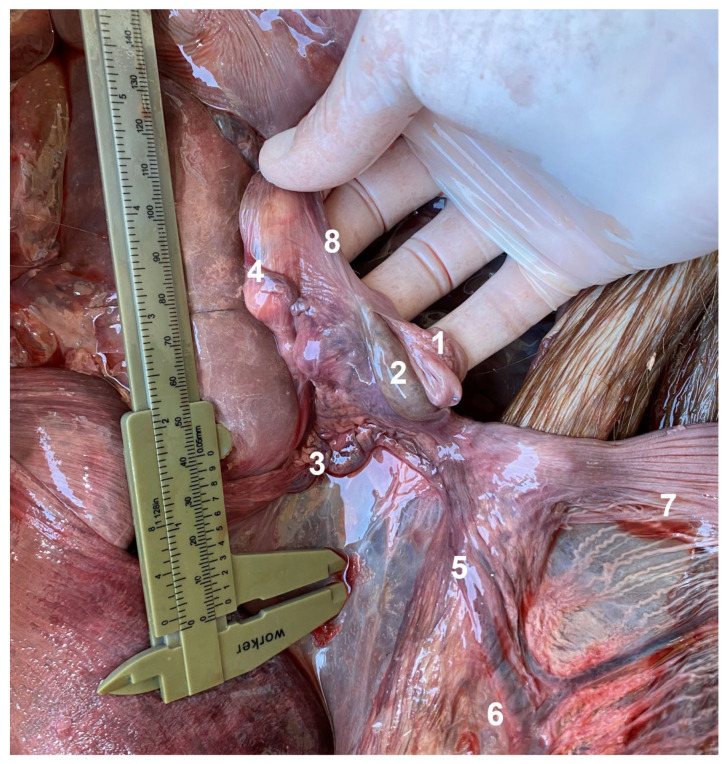
Vascular anatomy of reproductive organs, left approach. 1. uterine tube; 2. ovary; 3. ovarian vein giving rise to the tubal branch (4) and uterine branch (5) that anastomoses with the uterine vein (6); 7. left uterine horn; 8. suspensory ligament of the ovary.

**Figure 10:**
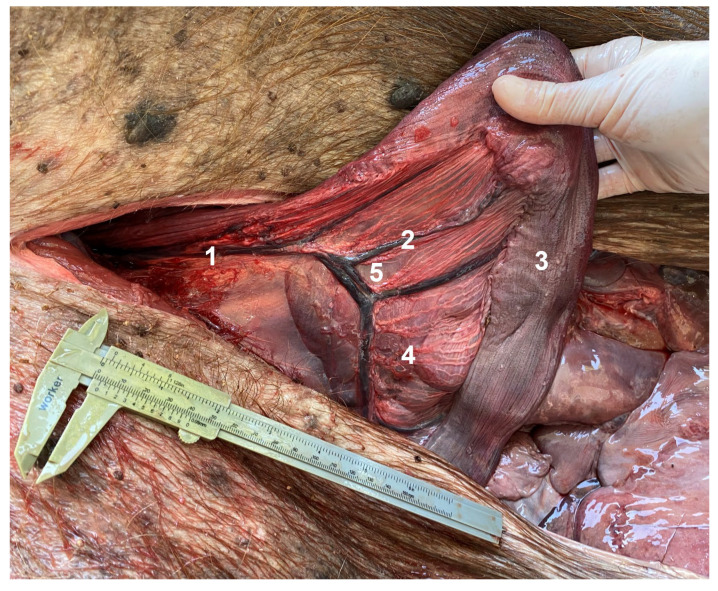
Ventral approach. Incision cranial to the pubis; caudal to the left and cranial to the right. 1. Uterine vein in a caudocranial direction; 2. branch of 1. to the uterine horn; 3. uterine horn; 4. branches of the uterine artery to the uterine horn. Note the comb aspect of the vessels on the mesometrium (5).

A minilaparotomy by this approach (incision length of approximately 3.5 cm) provided good exposure of both uterine horns and allowed the performance of Passos Nunes uterine horn ligature, also permitting hysterectomy performance in pregnant females. In the case of hysterotomies in pregnant multiparous females (when in possession of a more distensible uterus), with highly advanced pregnancy, the incision might be enlarged 4 cm cranially and the caudal part of the uterine tube might be identifiable through this approach. The irrigation of the uterine body and uterine horns arises cranially from a uterine branch from the ovarian artery and anastomoses caudally with the uterine artery, itself an internal iliac artery branch. The mesometrium is richly vascularized, with its arteries and vessels are distributed like a comb irrigating the uterine horns; thus, performing a hysterectomy becomes a challenge (Figure 7, Figure 8, Figure 9 and Figure 10).

### 3.3. Ventral Abdominal Ultrasound

**Figure 11:**
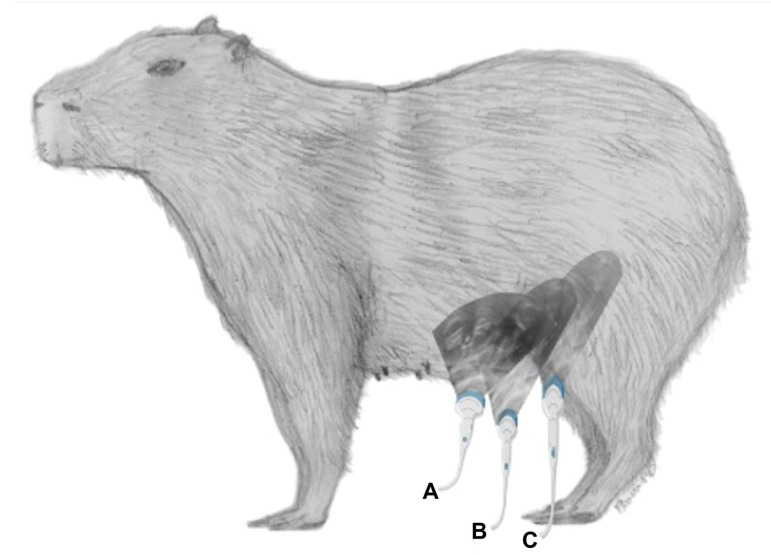
Schematic drawing showing the abdominal regions encompassed by ultrasonography: **A**. pre-umbilical region; **B**. umbilical region; **C**. post-umbilical region. Image created in BioRender.com by Flavia Maria Pia Montenegro Donoso.

**Figure 12:**
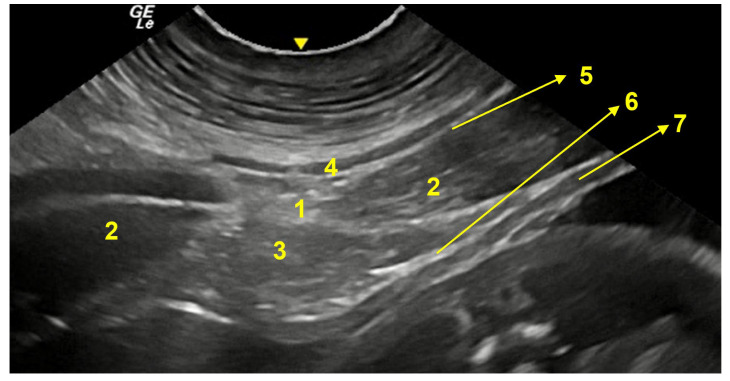
Transversal image of the pre-umbilical region (**A**). 1. linea alba; 2. rectus abdominis muscle; 3. falciform fat; 4. cutaneous muscle; 5. profound fascia together with aponeurosis of external abdominal oblique muscle; 6. transversus abdominis muscle together with parietal peritoneum; 7. uterine horn wall. Below, fetuses within 110 days of gestation.

**Figure 13:**
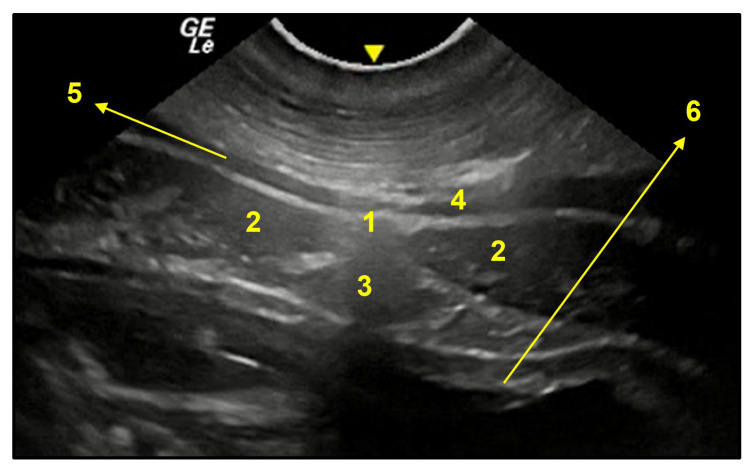
Transversal image of the umbilical region (B). 1. umbilical scar; 2. rectus abdominis muscle; 3. falciform fat; 4. cutaneous muscle; 5. profound fascia together with aponeurosis of external abdominal oblique muscle; 6. uterine horn wall.

**Figure 14:**
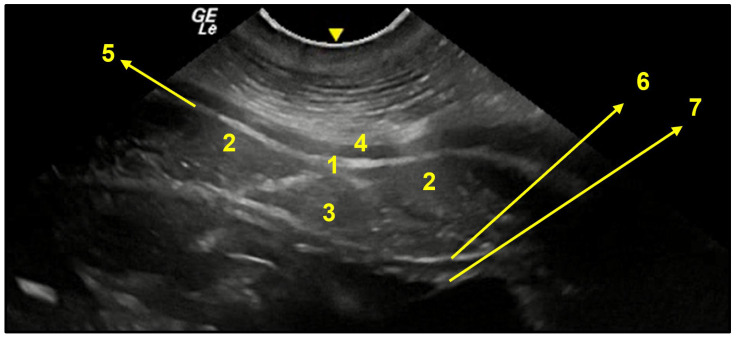
Transversal image of the post-umbilical region (**C**). 1. linea alba; 2. rectus abdominis muscle; 3. falciform fat; 4. cutaneous muscle; 5. profound fascia together with aponeurosis of abdominal external oblique muscle; 6. transversus abdominis muscle aponeurosis together with parietal peritoneum; 7. uterine horn wall.

The ultrasound findings are described in Figure 11, Figure 12, Figure 13 and Figure 14:

## 4. Discussion

In this study the lateral and ventral abdominal anatomy of female capybaras has been described to promote better surgical procedures for sterilization surgeries. The anatomy of the capybaras’ abdominal wall followed the general stratigraphy described for mammals [21], with variations concerning the thickness of the skin and cutaneous muscle as well as the distribution of muscular aponeurosis and facility of linea alba identification with a ventral approach, when compared to dogs and horses [22,23]. In agreement with our findings, the abdominal muscles of guinea pigs (*Cavia porcellus*), hystricomorph rodents closely related to capybaras, also encompass the external abdominal oblique muscle as the outermost layer, followed by the internal abdominal oblique muscle, transversus abdominis, and rectus abdominis [24], although we failed to find a detailed description of aponeurosis distribution and *linea alba* visibility in this species. The aponeurosis of external abdominal oblique muscle in capybaras followed the description of Risk [25] for Equidae, Bovidae, and primates. Despite the difficult identification of the linea alba in some of the post-umbilical macroscopy dissections performed here, we could confirm its presence in the pre- and post-umbilical regions through ultrasound images, corroborating the anatomical findings in eutherian mammals [25], although it might still be a challenge for the surgeon to find it during median post-umbilical celiotomy.

The superficial cutaneous muscle, also called the cutaneous maximus by Potter [24], has been described as occupying the lateral surface of the thorax and abdomen. Langworthy [26] described it as a thin layer of muscle lying just beneath the skin, of very variable extent, and considered it as a derivative of pectoral musculature whereas Bahri [27] describes it as a dermal striated muscle prevalent in lower mammals, including rodents, located within the subcutaneous layer of the skin. This muscular layer does not participate in *linea alba* formation and might cause extra bleeding on both surgical approaches to its thickness, adding an extra barrier to be sutured.

Entering the abdominal cavity, the cecum occupies the greatest part of the ventral abdomen, as described by Vazquez, Yanai, and Herrera [10,14,28] and no great omentum could be seen covering the abdominal viscera on its ventral surface during our dissections, corroborating Yanai [10] findings. The great cecum’s mobility and distention capacity allow it to be touched through a celiotomy or laparotomy depending on its distention or uterine distention, according to the pregnancy stage. Post-mortem tympanism might be responsible for cecum distention over the right antimere, favoring its syntopy with the right uterine tube. Photos of the syntopy between the organs were not realized because of the need to remove the intestines for visualization of the reproductive organs. The anatomy of the reproductive system described here followed the descriptions of Carvalho, Miglino, Pradere, Orihuela, and Mones y Ojasti [2,19,29,30,31] for female capybaras. The flank approach using a longitudinal minilaparotomy incision just caudal to the last rib and ventral of the sublumbar musculature provided rapid identification and traction of the ipsilateral ovary, permitting easy exposure of the uterine tube for surgical ligation/removal performance. The same axis of incision was used by Bester [32] for salpingectomy in Sprague Dawley rats *(Rattus norvegicus*). Yanai [10] in turn opted for a transverse incision in a dorsoventral direction (2 to 3 cm in length) toward the midline in female capybaras, performed at the midpoint of the distance between the wing of the ilium and the last rib, with this being the same axis and place of incision used by McGrath [33] for dogs and cats. We believe the axis of incision will not add to visualization of contralateral reproductive structures, demanding in any case a bilateral flank incision for tubal procedures, as confirmed by Yanai [10]. In relation to a ventral midline approach, Risk [25] reports a complex disposition of abdominal muscles’ aponeurosis in eutherian mammals with multiple sliding layers that could be immobilized after incision of the linea alba and suggests a parallel incision for median celiotomy. Considerations about muscular biomechanics and tissue repair were not covered in this study, making future studies necessary. The lack of sufficient data about the surgical anatomy for contraceptive surgery in female capybaras limits the comparison of the data presented here. For more accurate results of the syntopy of abdominal visceral with the reproductive organs, more cadavers should be dissected using the same technique of preservation, considering the great mobility of the cecum after post-mortem tympanism.

## 5. Conclusions

The stratigraphy of the abdominal wall of capybaras followed the general pattern described for other mammals. A bilateral laparotomy or midline post-umbilical celiotomy provided access to the reproductive organs of capybaras. While a bilateral laparotomy is necessary for good exposure of uterine tubes, a single midline celiotomy in a post-umbilical region provided good exposure of uterine horns but no access to the ovaries or uterine tubes, making the flank approach better for tubal ligation/removal and the post-umbilical approach for Passos Nunes uterine horn ligature, corroborating with our hypothesis.

## Data Availability

Not applicable.
